# Interferon-Inducible Transmembrane Protein 3-Containing Exosome as a New Carrier for the Cell-to-Cell Transmission of Anti-*Brucella* Activity

**DOI:** 10.3389/fvets.2021.642968

**Published:** 2021-03-16

**Authors:** Jihai Yi, Yueli Wang, Huan Zhang, Xiaoyu Deng, Jing Xi, Honghuan Li, Ningning Yang, Zhongchen Ma, Yong Wang, Chuangfu Chen

**Affiliations:** ^1^College of Animal Science and Technology, Shihezi University, Shihezi, China; ^2^Key Laboratory of Control and Prevention of Animal Disease, Xinjiang Production & Construction Corps, Shihezi, China

**Keywords:** *Brucella*, exosome, label-free proteomic analysis, IFITM3, immunity, intracellular survival

## Abstract

Exosomes are small extracellular vesicles that are released from cells and that function in intercellular communication. Recently, interferon-inducible transmembrane protein 3 (IFITM3) has been identified as a highly effective anti-intracellular pathogen protein that can inhibit the invasion of a wide range of pathogenic microorganisms. However, whether *Brucella* infection induces secretion of exosomes and whether these exosomes contain IFITM3 protein remain unknown. Here, we focused on the immune function of extracellular IFITM3 protein in the process of *Brucella* infection. This study is the first to show that *Brucella melitensis* strain M5 (*Brucella* M5) can stimulate macrophages to secrete large amounts of exosomes. Most importantly, we identified exosomes from *Brucella* M5-infected cells that were rich in molecules of IFITM3, and these exosomes could transmit the IFITM3 from one cell to another, thereby effectively inhibiting the intracellular survival of *Brucella*. Moreover, immunization with exosomes carrying IFITM3 decreased mouse spleen tissue damage and spleen colony forming unit (CFU), leading to the establishment of an anti-*Brucella* state in mice. In conclusion, our findings provide new insights into the anti-*Brucella* mechanism of IFITM3-containg exosomes, thus providing a theoretical foundation for systematic elaboration of the mechanisms of *Brucella* infection and host immunity. The results provide new ideas for the development of candidate vaccines for *Brucella*.

## Introduction

*Brucella* is a genus of Gram-negative facultative intracellular bacteria that cause serious damage to domestic animals and humans (1, 2). In animals, brucellosis predominantly causes chronic epididymitis in males and abortions and sterility in females, resulting in serious economic losses in livestock husbandry production (3). In humans, brucellosis causes joint pain, undulant fever, and fatigue. Studies have confirmed that *Brucella* has evolved a high-level immune escape strategy so that the host immune system cannot completely eradicate the bacteria; the pathogen can thus eventually establish a chronic infection (4). At present, it is believed that *Brucella* infection can stimulate the innate immunity of the body (5). Macrophages, as an important component of the innate immune system, are the key cells in defense against pathogen invasion (6). Studies have shown that the activation of macrophages plays an important role in clearing and controlling *Brucella;* 90% of the invading *Brucella* can be killed by eventual fusion with lysosomes (7). Therefore, understanding the relationship between *Brucella* infection and innate immunity and the interaction among immune cells is of great significance for controlling the spread of brucellosis.

Exosomes are small membrane-bound vesicles with diameters ranging from 30 to 120 nm. Exosomes are formed in the endosomal compartment by inward budding of endosomal membranes that are produced in late endosomal intracellular multivesicular bodies (8) (MVBs). After the fusion of MVBs with the plasma membrane, pools of exosomes accumulated in MVBs are released into the extracellular space (9). Those small vesicles can carry proteins, nucleic acids, and lipids to neighboring cells and thus modulate many biological processes such as cell growth, immune regulation, and cell migration (10–12). At present, research on the role of exosomes in the host immune response mainly focuses on tumor immunity, autoimmunity, and viral infection, while there are few studies on the role of exosomes in bacterial infections (especially intracellular bacteria). A recent study reported the protein profiles of extracellular vesicles-derived macrophages with *Mycobacterium tuberculosis* (Mtb) analyzed by label-free liquid chromatography–mass spectrometry (LC-MS)/MS quantitation technology. A total of 287 protein groups were identified in these vesicles, including host immune proteins and tuberculosis virulence-related proteins (13), and the host proteins played an important role in innate immunity (14). However, it is unknown whether *Brucella*, an intracellular bacterium, can also induce macrophages to produce exosomes.

Interferon-inducible transmembrane protein 3 (IFITM3) belongs to the family of IFN-stimulated genes (ISGs) (15) that are key antiviral effectors of the host innate immune system (16). Research has indicated that IFITM3 is also a major immune molecule in the host defense against Mtb infection (17). The reduction of pH value in lysosomes of host cells mediated by IFITM3 can effectively inhibit the intracellular proliferation of Mtb, suggesting that IFITM3 not only participates in antiviral immunity but also plays a crucial role in inhibiting the survival of intracellular bacteria (17, 18). The results from the study of Zhu have shown that IFITM3-containing exosomes were present in the extracellular microenvironment, and that study also determined that these exosomes could transmit the IFITM3-mediated antiviral activity from *Dengue* virus infected-cells to uninfected cells (19), revealing a new mode of antiviral defense. However, in the process of *Brucella* infection, whether IFITM3-containing exosomes have notable anti-intracellular bacterial activity in hosts is unknown.

This study is the first to extract and identify exosomes from the extracellular environment of *Brucella* M5-infected macrophages. Label-free proteomics analysis of such exosomes indicated that immune molecules of IFITM3 were enriched in the exosomes. Subsequently, we focused our investigation on the function of the IFITM3-laden exosomes *in vivo* and *in vitro*.

## Materials and Methods

### Cells, *Brucella* Strains, and Mice

*Brucella* M5 was provided by the Center for Chinese Disease Prevention and Control (Beijing, China). *Brucella* M5 was cultured in tryptone soya agar (TSA) or tryptone soya broth (TAB) at 37°C. Macrophages RAW264.7 were obtained from the Type Culture Collection of the Chinese Academy of Sciences (Shanghai, China). The macrophages were cultured in a 5% CO_2_ atmosphere in DMEM containing 10% exosome-depleted fetal bovine serum (FBS). Six-week-old BALB/c mice were purchased from the Xinjiang Medical University. All animal care in this experiment was performed in compliance with institutional animal care guidelines and relevant laws.

### *Brucella* M5 Infection of Macrophages RAW264.7

Macrophages RAW264.7 were infected with *Brucella* M5 for 1 h; the multiplicity of infection (MOI; bacteria:cell) was 100:1. Cells were incubated for 60 min. The macrophages were then washed three times with phosphate-buffered saline (PBS). Next, gentamicin (25 μg/ml) was added to the cell culture medium and incubated for 45 min to kill bacteria outside of the cells. The culture medium was discarded and replaced. After 24 h post-*Brucella* infection, the supernatants were collected from each plate.

### Isolation and Identification of Exosomes

Exosomes from each of the culture supernatants above were obtained by differential centrifugation. Briefly, cell supernatants were centrifuged at 1,000 *g* for 5 min, 5,000 *g* for 5 min, 14,000 *g* for 30 min, and 120,000 *g* for 1 h, followed by two wash with PBS buffer and purification to eliminate contaminating proteins by centrifugation at 120,000 *g* for 1 h. The purified exosomes were identified by nanoparticle tracking analysis (NTA), transmission electron microscopy (TEM), and Western blotting as described by Mitchell (20). The total protein concentration of exosomes was measured using the BCA Assay Kit (Pierce, Rochford, IL). Before the experiment, all isolated exosomes were stored at −80°C.

### Label-Free Analysis

In this experiment, the identified exosomes were sent to Applied Protein Technology Co., Ltd (Shanghai., China) for label-free quantitative proteomics analysis. The exosome samples were divided into two groups: one group comprised of exosomes released from normal macrophages RAW264.7 (uninfected group), and the other contained exosomes released from *Brucella-*infected macrophages RAW264.7 (infected group), with three replicates per group.

### Interferon-Inducible Transmembrane Protein 3 Interference Vector and Identification of Stable Expression Cell Lines

The 293FT cells and macrophages RAW264.7 were transfected with lentiviral packaging plasmids LV1-IFITM3 (siIFITM3) designed by GenePharma (Shanghai, China) and helper plasmids (PRSV-Rev, pMDL, and PCMV-VSVG) by Lipofectamine 2000 (Invitrogen, OH, USA). The ratios of plasmids are listed in Table 1. All of the plasmids and macrophages were mixed thoroughly and cultured at 37°C in a 5% CO_2_ incubator. IFITM3 stable interference cells (siIFITM3-Mø) were generated. After incubation for 48 h, the cell lines were identified using fluorescence microscopy (Nikon, Japan) and RT-PCR (Table 2).

**Table 1:** Lentivirus packaging system.

**Group**	**Reagent**	**Dosage (μl)**
A	Buffer A	250
B	LV1-IFITM3	6
	PRSV-Rev	3
	pMDL	3
	PCMV-VSVG	3
	dd H_2_O	Supplement to 250

**Table 2:** RNA transcription system of reverse transcription-PCR (RT-PCR).

**Gene**	**Primer name**	**Primer (5^**′**^ → 3^**′**^)**	**Tm (^**°**^C)**
IFITM3	IFITM3-F	GTC TCGCTCCTGGAAGAT GGTG	61
	IFITM3-R	CATTTGCAG TGGCAAAGT GGAG	
Reference gene	GAPDH-F	GGTGAAGGTCGGTGAACG	58
	GAPDH-R	CTCGCTCCTGGAAGATGGTG	

### Western Blot Assays

The different pretreated cells were lysed by high-efficiency RIPA Lysate (Solarbio, China) and boiled for 10 min at 100°C in SDS buffer. The total proteins were separated using SDS-PAGE and then transferred onto nitrocellulose membranes (Millipore, MA, USA). The membranes were blocked with 5% non-fat milk (Thermo Scientific, MA, USA) and probed with rabbit anti-phospho-IFITM3 (dilution 1:2,000, EterLife, China) for 1 h at 37°C. The membranes were incubated with HRP-conjugated mouse anti-rabbit IgG (dilution 1:5,000, ZSGB-BIO, China) for 1 h at 37°C. Finally, membranes were washed and stained using the HRP-DAB kit (ZSGB-BIO, China). Protein synthesis was analyzed by an enhanced chemiluminescence system.

### Real-Time PCR

Total RNA was isolated from siIFITM3-Mø cells and reverse transcribed to cDNA, with GAPDH as a reference gene. RT-PCR was performed with SYBR-Green (Roche, Switzerland) along with the primers listed in Table 2. Data were analyzed using the comparative 2^−ΔΔCt^ method.

### Colony Forming Units (CFU) and Cytokine Production Assay

Exosomes released from the different pretreated cells were incubated with siIFITM3-Mφ cells for 8 h and then infected with *Brucella* M5 (the infection method was as described above) and incubated for 4, 12, 24, and 48 h post-infection. Then, the cells were lysed with 0.5 ml of 0.2% Tween 20 for 15–30 min on ice, followed by rinsing of each well with 0.5 ml of PBS. Viable bacteria were quantified by serial dilution in sterile PBS and plating on TSA, and then were incubated at 37°C for 72 h followed by colony counting. And the levels of IFN-α and IFN-β in the supernatants were measured with an ELISA Quantikine Mouse kit (R&D Systems, MN, USA). The data were obtained from three independent experiments.

### Immunization of Mice With Exosomes

A total of four immunization programs were formulated: exosomes from uninfected macrophages RAW264.7 (Exo), exosomes from *Brucella* M5-infected macrophages RAW264.7 (Exo-M5-IFITM3), exosomes from *Brucella* M5 infected siIFITM3-Mø cells (Exo-M5-siIFITM3), and a PBS group. Each immunization program included three mice, and each of the mice was immunized *via* the tail vein route with a final injection volume of 100 μl (0.3 μg/μl) (the injection dose was referred to Cheng and Schorey (21). Immunization was repeated three times at 5-day intervals.

### *Brucella* Challenge

Thirty-five days following the final vaccination with exosomes, each group of mice was challenged with *Brucella* M5 by subcutaneous injection (1 × 10^6^ CFU/mouse in 100 μl of PBS). After 1, 3, 5, and 7 weeks post-infection, three *Brucella*-infected mice were humanely sacrificed to remove and weigh the spleens. The spleen was put into a centrifuge tube containing PBS buffer, 1 ml of 0.2% Triton X-100, and several small steel balls, and then homogenized in a homogenizer. Then, 100 μl of the homogenate was diluted in sterile saline and plated onto TSA. The plates were incubated at 37°C, and bacterial colony counts were expressed as log_10_CFU per spleen.

## Results

### Characterization of Exosomes Secreted From *Brucella*-Infected Macrophages RAW264.7

**Figure 1:**
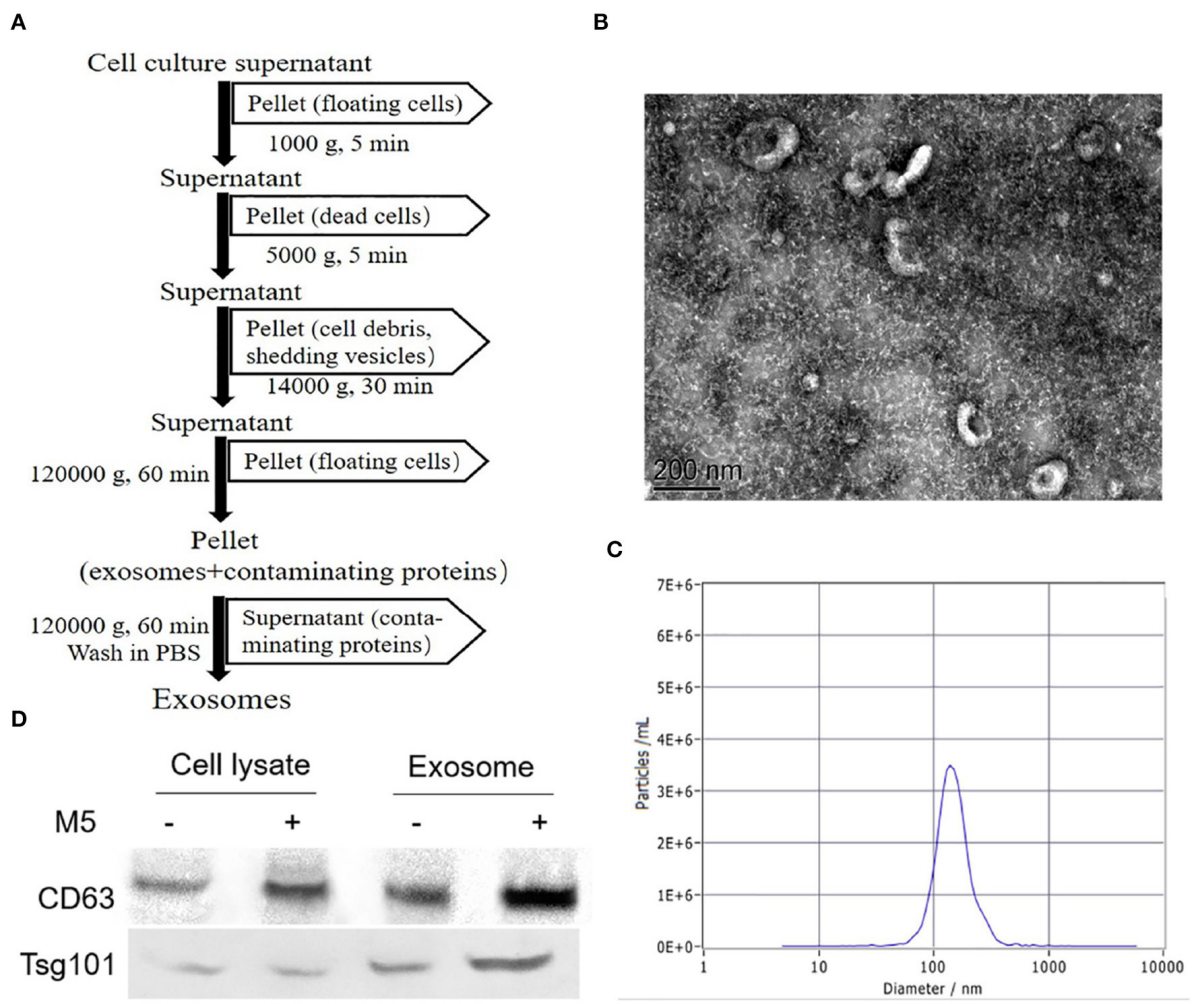
Purification and identification of exosomes secreted from *Brucella* M5-infected macrophages. **(A)** Method of the isolation and purification of exosomes from cell culture supernatant based on differential centrifugation. The purified exosomes secreted from *Brucella*-infected macrophages were analyzed by transmission electron microscopy (TEM). **(B)** The exosomes secreted from *Brucella*-infected macrophages size distributions were tested by nanoparticle tracking analysis (NTA) **(C)**. **(D)** Western blotting was used to identify exosomal markers (CD63, Tsg101) in exosomes (5 μg/well) derived from uninfected or *Brucella* M5-infected macrophages and in the corresponding whole cell lysates.

Exosomes released from *Brucella* M5-infected cells were analyzed by EM, NTA, and Western blotting. The results showed that these exosomes had a spherical cup-shaped appearance, with an average size of 40–120 nm (Figures 1A–C). In addition, comparison of macrophages RAW264.7 lysates with exosomal preparations indicated the presence of multiple specific exosome markers CD63 and Tsg101 (Figure 1D).

### Label-Free Proteomic Analysis of Exosomes Secreted From *Brucella*-Infected Macrophages RAW264.7

**Figure 2:**
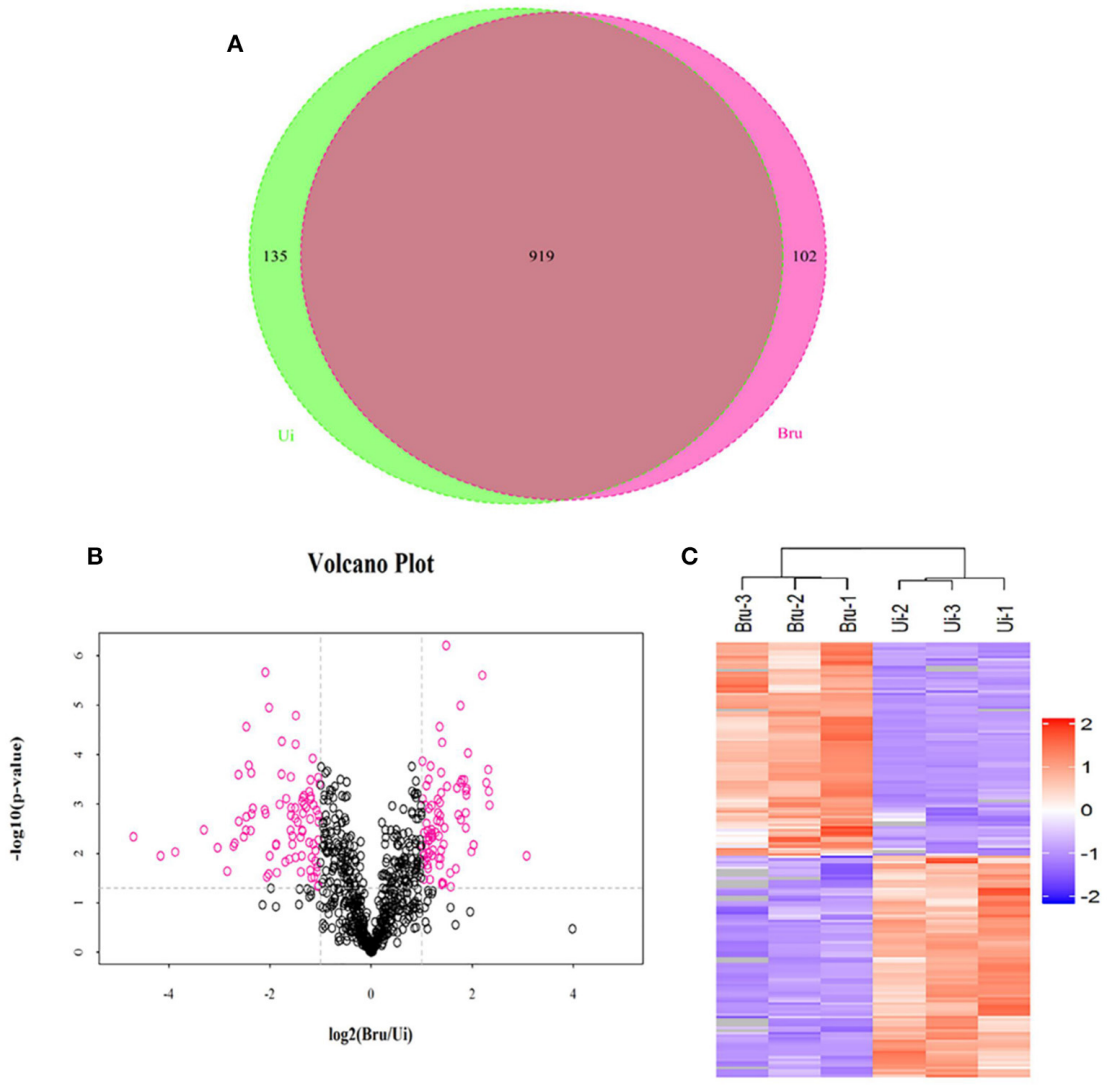
Proteomic profiling of exosomes derived from uninfected or *Brucella* M5-infected macrophages. **(A)** Venn diagram showing the overlap between exosome derived from *Brucella* M5-infected cells and uninfected cells. **(B)** Volcano plot and Heatmap **(C)** showing the differential protein expression of these two groups.

The aim of the analysis was to comprehensively explore the components of exosome-derived macrophages RAW264.7. Label-free quantitation technology was used to analyze the protein profiles of exosomes secreted from macrophages with or without *Brucella* infection. A total of 1,236 proteins were identified (Figure 2A), including 1,156 proteins derived from host cells and 80 proteins derived from *Brucella* M5. Among the 1,156 proteins, 89 proteins were found to be upregulated (Figures 2B,C), including interferon-induced transmembrane protein (IFITM3), lysozyme C-2 (Lyz2), and CD14 (Table 3). The functions of these proteins involved host immunity. This study focused on the biological functions of IFITM3. Based on the above results, we further explored the functions of IFITM3 in *Brucella* infection and host immunity.

**Table 3:** List of major protein expression differences in exosomes from host cells.

**Protein ID**	**Protein name**	**Gene name**	**Bru/Ui**	***T-*test *p-*value**	**state**
Q9CQW9	Interferon-induced transmembrane protein 3	IFITM3	4.44	0.0037	Up
P08905	Lysozyme C-2	Lyz2	3.30	0.0016	Up
P14211	Calreticulin (MHC-I)	Calr	2.61	0.0442	Up
Q9Z0M5	Lysosomal acid lipase/cholesteryl ester hydrolase	Lipa	2.57	0.0022	Up
Q61937	Nucleophosmin	Npm1	2.56	0.0027	Up
P80316	T-complex protein 1 subunit epsilon	Cct5	2.26	0.0001	Up
P10810	Monocyte differentiation antigen CD14	CD14	2.23	0.0119	Up
Q9Z0X1	Apoptosis-inducing factor 1, mitochondrial	Aifm1	2.21	0.0045	Up
O09159	Lysosomal alpha-mannosidase	Man2b1	2.19	0.0003	Up
P15379	CD44 antigen	CD44	2.16	0.1230	Up
P26151	High affinity immunoglobulin gamma Fc receptor I	Fcgr1	2.12	0.0052	Up
P41731	CD63 antigen	Cd63	2.08	0.0329	Up
Q8CIH5	phospholipase C-gamma (1)	Plcγ2	2.01	0.0112	Up
Q99MN9	Propionyl-CoA carboxylase beta chain, mitochondrial	Pccb	2.00	0.0378	Up
Q8VBV7	COP9 signalosome complex subunit 8	Cops8	0.49	0.0011	Down
P40237	CD82 antigen	Cd82	0.49	0.0180	Down
P51863	V-type proton ATPase subunit d 1	Atp6v0d1	0.45	0.0298	Down
P57746	V-type proton ATPase subunit D	Atp6v1d	0.43	0.0003	Down
P61957	Small ubiquitin-related modifier 2	Sumo2	0.47	0.0092	Down
Q9CZ04	COP9 signalosome complex subunit 7a	Cops7a	0.24	0.0111	Down
Q03265	ATP synthase subunit alpha, mitochondrial	Atp5f1a	0.38	0.0035	Down

### Silencing the IFITM3 Gene Was Beneficial to the Intracellular Survival of *Brucella* M5

**Figure 3:**
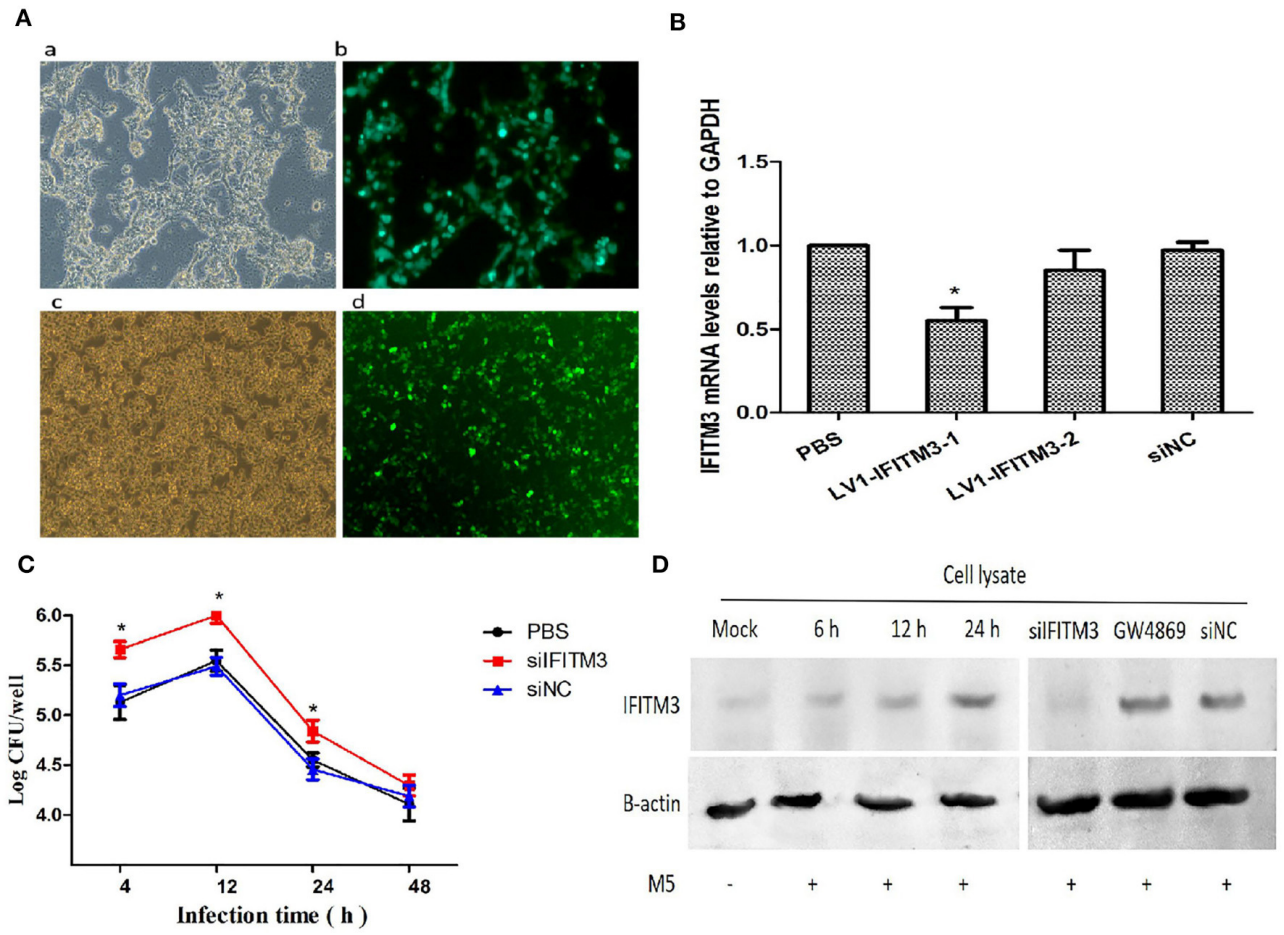
IFITM3 protein is required for the establishment of host immune response against *Brucella*. **(A)** LV1-IFITM3 packaging and infection process. (a): 293T cells; (b): Lentivirus package; (c): RAW264.7 cells; (d): Lentivirus transfection. **(B)** RT-PCR analysis of relative mRNA expression of IFITM3 in macrophages infected with the siRNA-IFITM3 lentivirus vectors. The 2^−ΔΔCt^ method was used to calculate relative gene expression. **(C)** *Brucella* intracellular growth was determined after 4, 12, 24, and 48 h post-infection by CFU test. **(D)** Macrophages pretreated with IFITM3 siRNA, siNC, or GW4869 were infected with *Brucella* M5 and were lysed after 24 h post-infection. The IFITM3 in cell lysate was analyzed by Western blotting. All treatments were repeated three times with *n* = 3/time point. Statistical significance is indicated by *p < 0.05.

To understand the role of endogenous IFITM3 in the interaction between *Brucella* and host macrophages, we first utilized siRNA technology to successfully silence the expression of the IFITM3 gene in macrophages RAW264.7 (Figures 3A,B). After infection with *Brucella* M5 at different time points, Western blot analysis showed that *Brucella* M5 infection could induce the activation of IFITM3 in a time-dependent manner, reaching the highest value at 24 h. Moreover, the expression of IFITM3 was inhibited in siIFITM3-Mø cells. Adding GW4869, a specific inhibitor of exosome release (22), or an empty interference vector did not affect IFITM3 expression (Figure 3D). IFITM3-silenced macrophages infected with *Brucella* were lysed, and the CFU results showed that compared with the PBS control group, silencing the IFITM3 gene significantly promoted the intracellular survival of *Brucella* M5 (*p* < 0.05) in a time-dependent manner. However, the empty interference vector did not affect the intracellular survival of *Brucella* M5 (Figure 3C). These results suggest that IFITM3 expression in macrophages contributes to host cell resistance to *Brucella* infection.

### IFITM3 Can Be Released to Extracellular Space v*ia* the Exosomal Pathway

**Figure 4:**
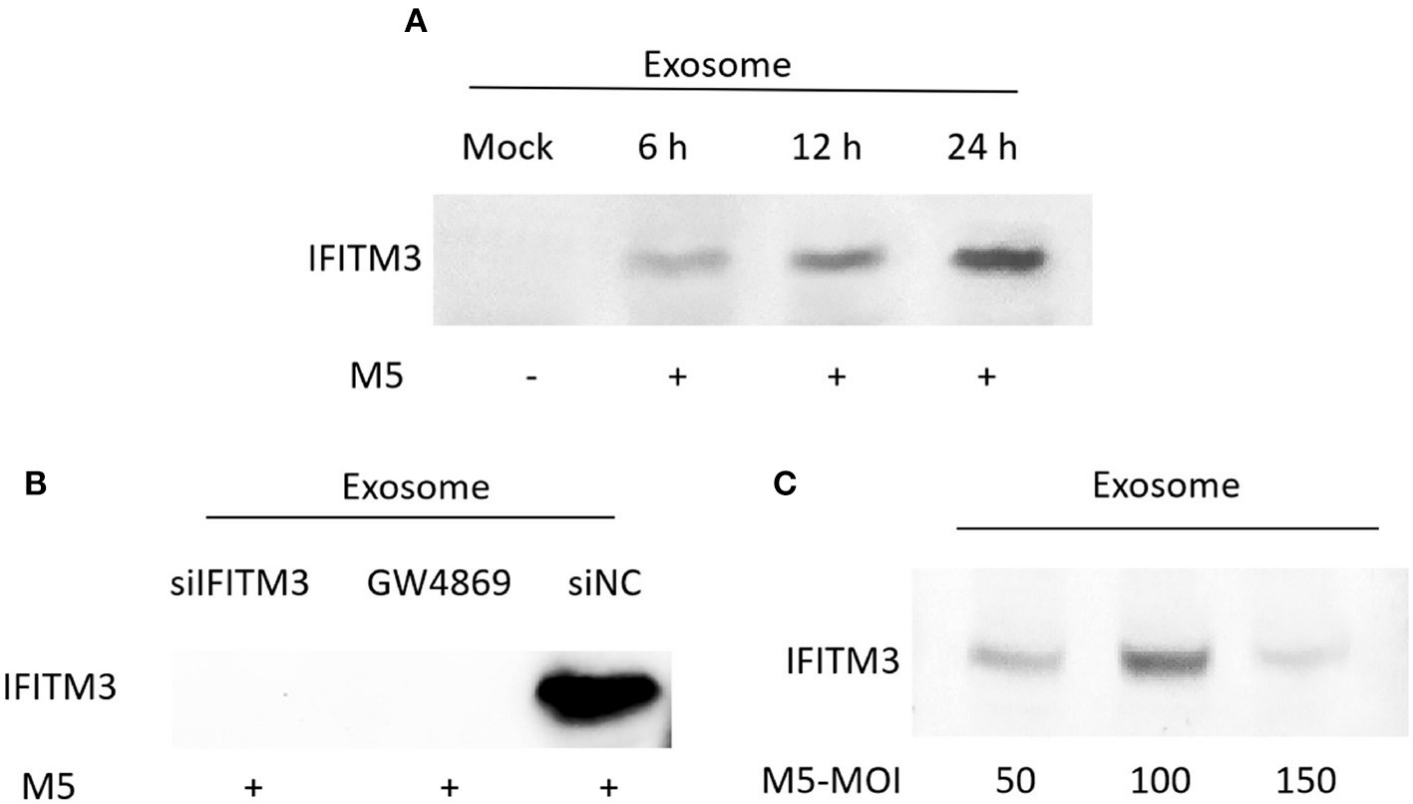
Identification of IFITM3 in exosomes secreted from *Brucella* M5-infected cells. **(A)** Macrophages were infected with *Brucella* M5 for 6, 12, and 24 h, and exosomes in the cell culture media were extracted. Western blot analysis of IFITM3 protein in exosome. **(B)** Macrophages pretreated with IFITM3 siRNA, siNC, or GW4869 were infected with *Brucella* M5, and exosomes derived from each group were extracted. Western blot analysis of IFITM3 protein in exosome. **(C)** Macrophages were infected with *Brucella* M5 at different MOI, and exosomes in this cell culture medium were extracted. Western blot analysis of IFITM3 protein in exosomes.

To determine whether exosomes derived from *Brucella* M5-infected macrophages RAW264.7 were able to transfer immunocompetent IFITM3 molecules to the extracellular space, we extracted exosomes in cell culture supernatants at different time points of *Brucella* M5 infection. The results showed that exosomes derived from macrophages RAW264.7 contained a high abundance of IFITM3 in a time-dependent manner, reaching a maximum at 24 h post-infection (Figure 4A). However, as shown in Figure 4B, there was no IFITM3 protein inside exosomes derived from *Brucella*-infected siIFITM3-Mø cells. Furthermore, different multiplicity of infection (MOI) levels affected the ability of exosomes to load IFITM3, reaching the highest loading capacity with an MOI of 100 (number of bacteria: number of cells = 100:1) (Figure 4C). Taken together, our data demonstrate that IFITM3 can be exported from macrophages by the exosomal pathway.

### IFITM3-Containing Exosomes Are Internalized by Macrophages

**Figure 5:**
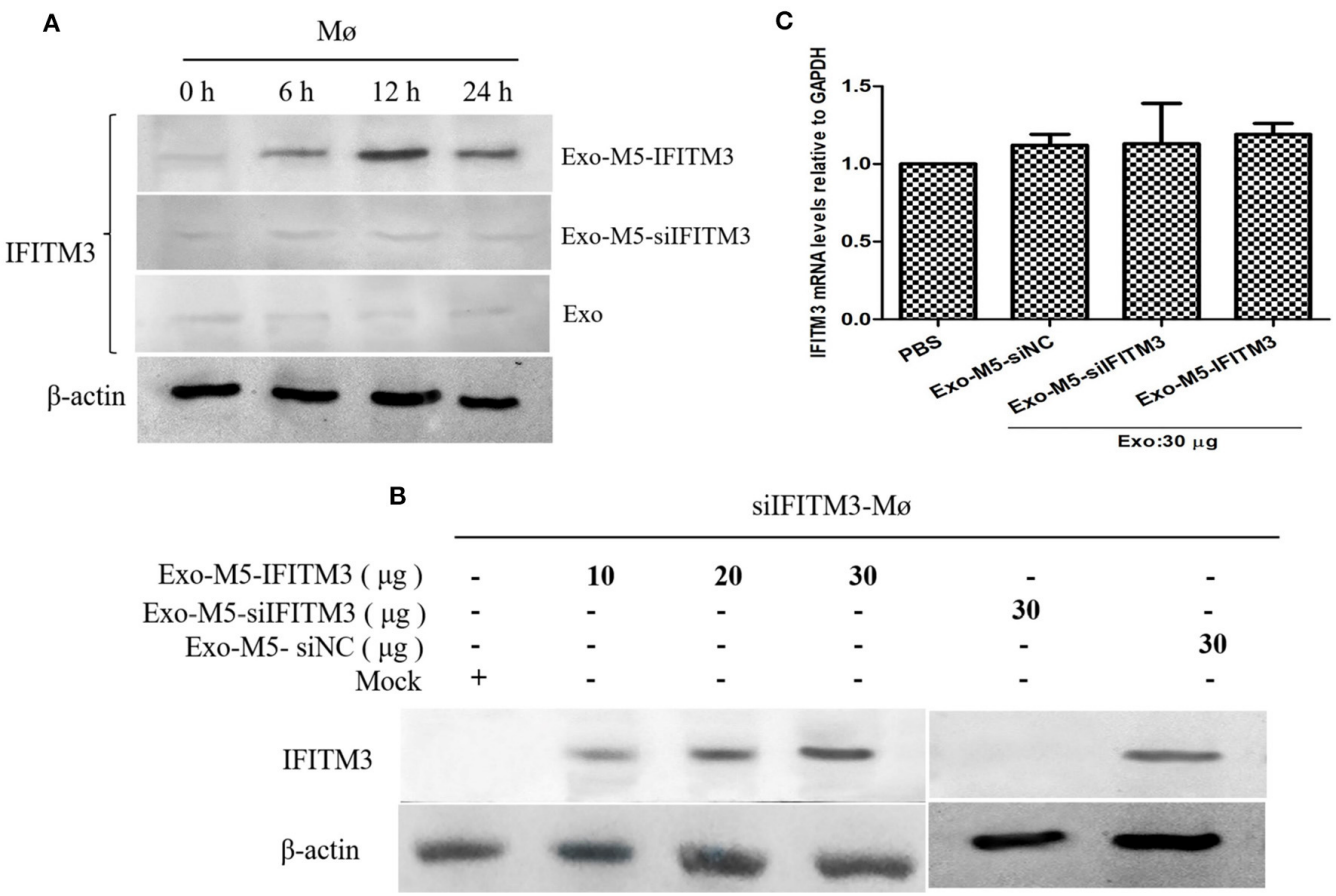
Exosomes released from *Brucella* M5-infected cells transfer IFITM3 to recipient cells. **(A)** Macrophages were incubated with purified 30 μg/ml of Exo-M5-IFITM3, Exo-M5-siIFITM3, or Exo for 0, 6, 12, and 24 h. Western blot analysis of IFITM3 protein in these cell groups. **(B)** siIFITM3-Mø cells were incubated with different doses of purified exosomes (Exo-M5-IFITM3, Exo-M5-siIFITM3, and Exo-M5-siNC), Western blot analysis of IFITM3 protein in these cell groups. **(C)** The transcription level of IFITM3 in normal macrophages from each group as analyzed by RT-PCR; the expression levels of this protein were normalized to that of GAPDH.

The finding above, that exosomes derived from *Brucella*-infected macrophages carried a high abundance of IFITM3 and were released into the extracellular space, prompted us to explore whether the IFITM3-laden exosomes were internalized by other host macrophages in the immune system. If true, this would mean that the anti-*Brucella* activity could be transferred to the target macrophages. In this study, we investigated such a possibility by incubating macrophages RAW264.7 pretreated with exosome inhibitor GW4869 with exosomes collected from *Brucella* M5-infected cells (Exo-M5-IFITM3), *Brucella*-infected siIFITM3-Mø cells (Exo-M5-siIFITM3), or normal cells (Exo). As shown in Figure 5A, a significant increase in the abundance of IFITM3 was observed in the target macrophages incubated with Exo-M5-IFITM3 (30 μg/ml), and the maximum value was reached at 12 h post-incubation. However, the incubation of Exo-M5-siIFITM3 and Exo hardly affected the protein abundance of IFITM3 inside target macrophages. In addition, detection of IFITM3 proteins in the lysates of siIFITM3-Mø cells treated with Exo-M5-IFITM3 for 12 h, as determined by Western blotting, was dose-dependent, reaching the highest value at 30 μg/ml. Interestingly, 30 μg/ml of Exo-M5-siIFITM3 did not increase the abundance of IFITM3 protein in the siIFITM3-Mø cells (Figure 5C). Furthermore, in order to confirm that the increased IFITM3 protein abundance in the recipient macrophages was due to IFITM3-laden exosomes instead of a stimulated expression of endogenous IFITM3 genes in the recipient cells, we measured the IFITM3 mRNA transcription levels in normal macrophages pretreated with Exo-M5-siIFITM3, Exo-M5-siNC, or Exo-M5-IFITM3 *via* RT-PCR (Figure 5B). The results showed that exosomes carrying or not carrying IFITM3 did not cause an increase of endogenous IFITM3 in the receptor macrophages. Taken together, these results demonstrated that exosomes carrying IFITM3 protein can be internalized by recipient macrophages, thereby resulting in an increase in the abundance of the IFITM3 protein.

### The Anti-*Brucella* Activity of Internalized IFITM3-Containing Exosomes

**Figure 6:**
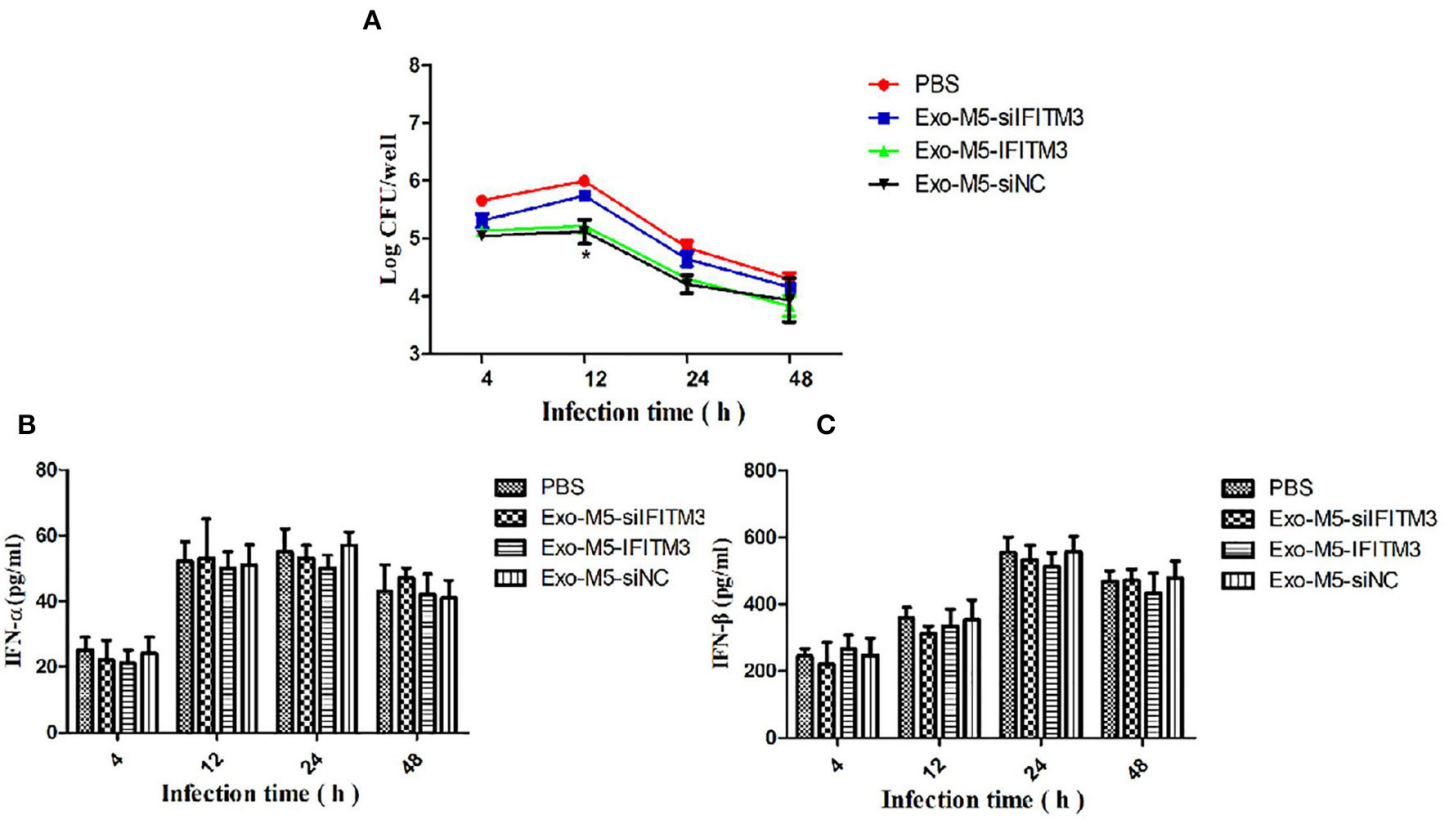
The anti-*Brucella* activity of IFITM3-laden exosomes. siFITM3-Mø cells incubated with PBS, Exo-M5-siIFITM3, Exo-M5-IFITM3, or Exo-M5-siNC were infected with *Brucella* M5. The CFU level was measured at different time points post-infection. **(A)** The intracellular bacterial survival was tested by measuring CFU, with PBS as a control group. The macrophage culture supernatants were harvested and IFN-α **(B)** and IFN-β **(C)** production (pg/ml) were assessed using the ELISA assay. All treatments were repeated three times with *n* = 3/time point. Statistical significance is indicated by *p < 0.05.

To investigate whether IFITM3-containing exosomes (Exo-M5-IFITM3) internalized by recipient macrophages could affect intracellular survival of *Brucella*, siFITM3-Mø cells were cultured in DMEM containing 10% exosome-depleted FBS and exposed to 30 μg of purified Exo-M5-IFITM3 for 12 h. After *Brucella* M5 infected the exosome-treated cells, CFU counts were performed at different time points post-infection. As shown in Figure 6A, compared with the PBS control group, the number of intracellular bacteria in recipient cells pretreated with Exo-M5-IFITM3 or Exo-M5-siNC significantly decreased after 4, 12, 24, and 48 h post-*Brucella* infection (*p* < 0.05), but the Exo-M5-siIFITM3 and PBS groups had no effect on the intracellular replication of *Brucella* M5. In order to verify the relationship between the release of type 1 interferon and the expression of IFITM3, ELISA results showed that Brucella M5 could induce macrophages to release IFN-β and IFN-α; however, IFITM3-exosomes treatment did not affect the expression of IFN-α (Figure 6B) and IFN-β (Figure 6C). Taken together, our data indicated exosome delivery of IFITM3 to recipient macrophages, thereby effectively inhibiting the intracellular survival of *Brucella* and conferring resistance to *Brucella* persistent infection. Interestingly, IFITM3-containing exosomes mediated the decrease in intracellular replication of *Brucella* was not due to the release of non-specific type I interferon.

### Interferon-Inducible Transmembrane Protein 3-Containing Exosomes Protected Mice Against *Brucella* M5 Infection

**Figure 7:**
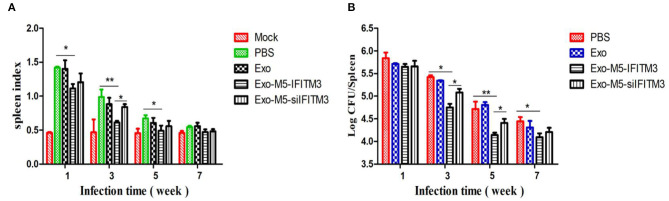
Mice immunized with exosomes carrying IFITM3 were protected against *Brucella* infection. Mice immunized with different groups of exosomes were infected with *Brucella* M5 for 1, 3, 5, and 7 weeks infection. Then, each group of mice was sacrificed, and mice spleen index **(A)** and spleen CFU **(B)** were measured. All treatments were repeated three times with *n* = 3/time point. Statistical significance is indicated by *p < 0.05, **p < 0.01.

To determine whether IFIMT3-containing exosomes could boost protective activity against *Brucella* infection, mice were given a dose of 1 × 10^6^ CFU with *Brucella* M5 after the final exosome immunization (35 days). Then, 1, 3, 5, and 7 weeks later, each group of three mice was sacrificed, and then the spleen index (spleen weight/body weight) and spleen CFU were calculated. As shown in Figure 7A, in the Exo-M5-IFITM3 group, the spleen index showed a significant increase compared with the Exo-M5-siIFITM3 group at 3 weeks of infection but was still lower than that of the PBS control group at 1, 3, and 5 weeks of infection (*p* < 0.05). The CFU test showed that the Exo-M5-IFITM3 group had a significantly higher spleen bacterial load compared to the Exo-M5-siIFITM3 group at 3, 5, and 7 weeks of infection, but was still lower than that of the PBS control group at 3 and 5 weeks of infection (*p* < 0.05) (Figure 7B). In addition, immunization of exosomes from uninfected macrophages RAW264.7 (Exo group) did not decrease the spleen index or spleen CFU. Thus, these results indicated that exosomes released from *Brucella* M5-infected macrophages could reduce *Brucella*-induced spleen tissue damage and provide protective activity against *Brucella* infection. IFITM3 contained in exosomes might be a key cellular restriction factor for *Brucella* infection.

## Discussion

Interferon-inducible transmembrane protein 3 is an efficacious restriction factor with broad-spectrum antiviral activity in host cells (23). However, an important finding of our present study, that antiviral molecules of IFITM3 can be transferred intercellularly from *Brucella*-infected macrophages to uninfected neighbors by extracellular exosomes and thereby confer resistance to *Brucella* persistent infection, provides new insights into the functional significance of antimicrobial induced by IFITM3. Our verification of this ability of IFITM3-laden exosomes may provide a foundation for further elucidating the molecular mechanism of *Brucella* infection and host innate immunity, eventually leading to the development of new candidate vaccines against *Brucella*.

Although IFITM3 is widely used in antiviral research, some studies had found that IFITM3 expression could enhance endosomal acidification in Mtb-infected monocytic cells, thereby directly inhibiting bacterial pathogen growth and implying that IFITM3 may be involved in the immune response against intracellular bacteria (24, 25). Several other intracellular pathogens, such as *Salmonella enterica* or *Yersinia pestis*, also depend on phagolysosome acidification arrest for survival and reproduction in host cells (26). Thus, IFITM3 may have a similar biological function during *Brucella* infection. This study found that *Brucella* M5 can indeed induce macrophages to express IFITM3 in a time-dependent manner. Silencing the IFITM3 gene was beneficial to the intracellular survival of *Brucella* M5. During our experiment, the interference efficiency of IFITM3 was always about 50%, which might be due to the type of host cell. But fortunately, the interference efficiency of 50% could also significantly affect the survival of Brucella *in vivo* and *in vitro*. It also indirectly indicated that IFITM3 protein played an important role in the process of resistance to bacterial reproduction. Accordingly, the degree of activation of IFITM3 affected the efficiency of bacterial intracellular survival in the process of *Brucella* infection.

Exosomes are important mediators of intercellular information exchange. Exosomes can transfer proteins, nucleic acids, and lipids to neighboring or distant cells, and thus help to regulate various physiological or pathological processes (27). Recent studies have found that Mtb infection can induce immune cells to release exosomes containing bacterial antigens and related immune factors. These exosomes play an important role in initiating the host natural immunity and acquired immune response (28). In this study, we also successfully isolated and identified *Brucella* M5-infected macrophage-derived exosomes for the first time. In order to further determine the protein composition of the exosomes derived from *Brucella*-infected cells, we compared the proteomes of exosomes released from *Brucella*-infected (*Bru*-inf-Exo) and uninfected macrophages using label-free quantitation technology. We found that 89 proteins were increased in abundance in *Bru*-inf-Exo, including several proteins associated with immune function, such as IFITM3, Lyz2, and CD14. IFITM3 had the highest protein abundance. Interestingly, IFITMT-laden exosomes might be considered as novel mediators in the transfer of antiviral effects from infected cells to uninfected cells (19). Therefore, we tested whether the exosomes carrying IFITM3 could transfer the anti-*Brucella* activity to other uninfected cells. The results demonstrated that IFITM3 protein induced by *Brucella* infection was mainly released into the extracellular space in the form of exosomes, and the ability to release IFITM3 was related to the number of infecting *Brucella*. According to (29) during the formation of exosomes, IFITM3 can be transferred into the multivesicular complex (MVB). Rab family members are involved in the formation and release of exosomes carrying IFITM3 (29). However, a high concentration of *Brucella*, as discovered by our present study, may interfere with the biological function of macrophages; this would impede exosomes from loading and transporting the IFITM3 protein. Because of the special membrane structure of exosomes, they can fuse with receptor cell membranes. We demonstrated here that exosomes carrying IFITM3 could be internalized by receptor cells, thereby increasing the abundance of exogenous IFITM3 protein in recipient cells. This in turn can effectively inhibit the intracellular survival of *Brucella* M5. This immune pathway appears to represent a new, attractive potential anti-intracellular bacteria strategy.

Animal vaccination is still the most effective way to control the spread of brucellosis (30). As a special immune mediator, exosomes have been used in the development of many new vaccines. One study found that exosomes derived from macrophages infected with Mtb provide protective immunity to mice, suggesting that exosomes might serve as a new cell-free vaccine or immunopotentiator against Mtb infection. ISGs have been demonstrated to be attractive and efficient anti-microbial effectors (31). Accordingly, it is of great clinical significance to use exosomes as a kind of nano-shuttle carrier for immune factors or drug delivery (32). In such a situation, IFITM3-laden exosomes may provide a direction for the development of new *Brucella* vaccines. It will be necessary to conduct small animal studies to test the feasibility and effectiveness of applying exosome-based IFITM3 delivery for anti-*Brucella* purposes in hosts. The spleen is the organ with the highest abundance of immune cells, and it is also the most active place for *Brucella* reproduction (33). Our results showed that IFITM3-laden exosomes could more effectively reduce spleen damage and spleen CFU during the process of *Brucella* infection in mice compared with exosomes lacking IFITM3, confirming that IFITM3-containing exosomes may be one of the main treatments to improve the immune response and inhibit the proliferation of *Brucella*. Previous studies demonstrated that exosomes released from macrophages infected with *Mycobacterium bovis* stimulated host cell production of antigen-specific IFN-γ and high levels of TNF-α and IL-12/p40 (34, 35). Consequently, further studies are needed to explore how IFITM3-containing exosomes improve the immune level and thereby inhibit persistent infection of *Brucella in vivo*, and to determine whether there are other immune molecules and antigens in exosomes involved in the activation of anti-*Brucella* immunity in mice.

In summary, our data provide the first evidence for the existence of an extracellular vesicle, an exosome carrying IFITM3, which can transmit anti-*Brucella* activity from one cell to another, thereby effectively inhibiting *Brucella* infection in the host. This conclusion provides a theoretical foundation for the systematic elaboration of the mechanism of *Brucella* infection and host immune response, and our findings have provided new ideas for the development of potential candidate vaccines for *Brucella*.

## Data Availability Statement

The raw data supporting the conclusions of this article will be made available by the authors, without undue reservation.

## Ethics Statement

The procedure of animal experiments was approved by the Institutional Animal Care and Use Committee of the First Affiliated Hospital of medical college, Shihezi Universityy (No. A201814901). Written informed consent was obtained from the owners for the participation of their animals in this study.

## Author Contributions

JY, YuW, and CC were responsible for designing experiments. JY, YoW, HZ, JX, and XD performed the experiments and assisted with the sample collections. JY and NY analyzed the data. JY, YuW, and ZM wrote and revised the manuscript. All authors read and approved the final manuscript.

## Conflict of Interest

The authors declare that the research was conducted in the absence of any commercial or financial relationships that could be construed as a potential conflict of interest.
